# Single-shot spectrometer using diamond microcrystals for X-ray free-electron laser pulses

**DOI:** 10.1107/S1600577522001205

**Published:** 2022-03-21

**Authors:** Ichiro Inoue, Eito Iwai, Toru Hara, Yuichi Inubushi, Kensuke Tono, Makina Yabashi

**Affiliations:** a RIKEN SPring-8 Center, 1-1-1 Kouto, Sayo, Hyogo 679-5148, Japan; b Japan Synchrotron Radiation Research Institute, 1-1-1 Kouto, Sayo, Hyogo 679-5198, Japan

**Keywords:** X-ray free-electron lasers, single-shot spectrometers, SACLA

## Abstract

A simple spectrometer using diffraction from diamond microcrystals has been developed to diagnose single-shot spectra of X-ray free-electron laser (XFEL) pulses. The large grain size and uniform lattice constant of the adopted crystals enable characterizing the XFEL spectrum at a resolution of a few eV from the peak shape of the powder diffraction profile. This single-shot spectrometer has been installed at beamline 3 of SACLA and is used for daily machine tuning.

## Abstract 2

A spectrometer using diamond microcrystals is proposed and demonstrated to diagnose the spectral properties of X-ray free-electron laser pulses.

## 1. Introduction

X-ray free-electron lasers (XFELs) based on the self-amplified spontaneous-emission scheme (Bonifacio *et al.*, 1984 ▸; McNeil & Thompson, 2010 ▸) are newly developed light sources that generate intense hard X-ray pulses with femtosecond duration. Due to the stochastic nature of the X-ray amplification processes and possible fluctuation of the electron-beam properties, each XFEL pulse has different spectral properties. Evaluating the spectrum is of great importance for optimizing machine parameters as well as interpreting data from experiments using XFEL pulses.

For measuring single-shot spectra of XFEL pulses, dispersive spectrometers have been intensively studied (Yabashi *et al.*, 2006 ▸; Inubushi *et al.*, 2012 ▸, 2017 ▸; Zhu *et al.*, 2012 ▸; Makita *et al.*, 2015 ▸; Rich *et al.*, 2016 ▸; Katayama *et al.*, 2016 ▸; Rehanek *et al.*, 2017 ▸; Grünert *et al.*, 2019 ▸; David *et al.*, 2021 ▸). An example of such spectrometers is one that combines a flat single-crystal analyzer and an elliptical mirror to increase the angular divergence of the X-ray beam (Yabashi *et al.*, 2006 ▸). The measurable spectral range and the energy resolution of the spectrometer can be varied by changing the reflection plane of the analyzer crystal. For example, the use of crystal planes with high reflection indices enables measurement of the high-resolution spectrum (resolution of ∼10 meV) at the cost of narrow spectral range (several eV), while one can measure the entire spectrum at a resolution of a few eV by selecting a crystal plane with low reflection index. Dispersive spectrometers using bent single crystals, which have been implemented at the LCLS (Zhu *et al.*, 2012 ▸; Rich *et al.*, 2016 ▸), European XFEL (Grünert *et al.*, 2019 ▸) and SwissFEL (David *et al.*, 2021 ▸), can also measure the entire spectrum at a resolution of a few eV. These spectrometers, however, require back-and-forth alignments of the components (the crystal angle and the mirror parameters/bending radius), causing complications in alignment and preventing the use of spectral information for daily machine tuning.

As a convenient tool to diagnose the spectral properties of each XFEL pulse, a wavelength monitor was introduced at SACLA (Ishikawa *et al.*, 2012 ▸; Tono *et al.*, 2013 ▸). This monitor measures X-ray diffraction from a 15 µm-thick nanocrystalline diamond foil and evaluates the central photon energy of each XFEL pulse from the position of the diffraction peak. The resolution of this monitor is limited to a few tens of eV due to the large angular spread of the diffraction peak originating from the small crystal size. Thus, more detailed spectral information, such as the photon-energy spread and the shape of the spectrum, is not accessible.

To facilitate characterization of the detailed spectral properties of XFEL pulses, we have developed a simple single-shot spectrometer using X-ray powder diffraction from diamond microcrystals and installed the spectrometer in experimental hutch 1 (EH1) of SACLA beamline 3 (BL3) (Tono *et al.*, 2013 ▸; Yabashi *et al.*, 2015 ▸). The concept of the new spectrometer, its energy resolution evaluated with monochromated XFEL pulses and application of the spectrometer to the machine tuning are described in the following.

## 2. Spectrometer using diamond microcrystals

**Figure 1:**
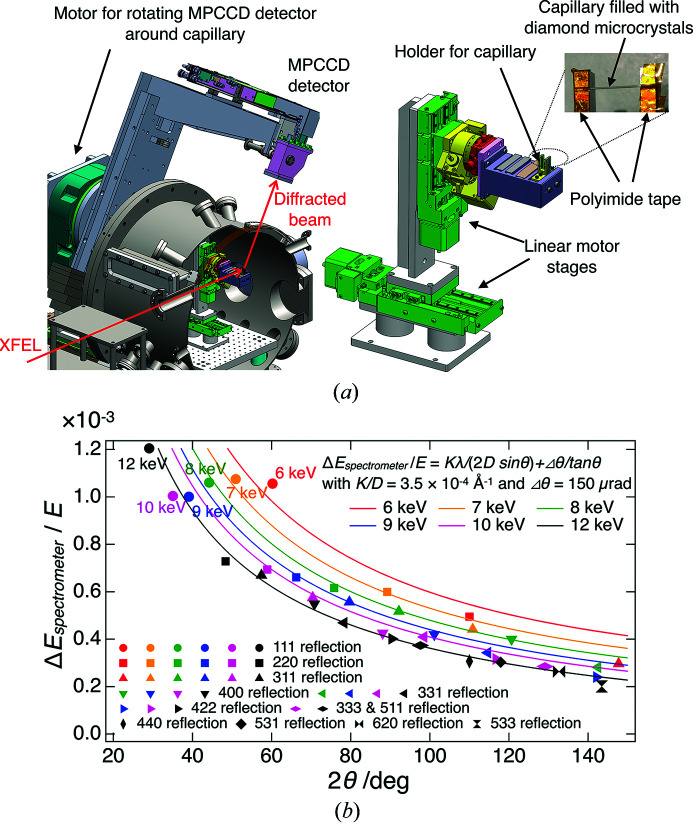
(*a*) A schematic of the spectrometer using diamond microcrystals in EH1 of SACLA BL3. (*b*) The energy resolution of the spectrometer evaluated with the monochromated XFEL pulses. Solid curves show expected energy resolution of the spectrometer for the case when *K*/*D* = 3.5 × 10^−4^ Å^−1^ and Δθ = 150 µrad.

Fig. 1 ▸(*a*) shows a schematic illustration of the new spectrometer in EH1 of SACLA BL3. The spectrometer consists of a two-dimensional detector with 50 µm square pixels [multi-port charge-coupled device (MPCCD) detector (Kameshima *et al.*, 2014 ▸)], and a 300 µm-diameter capillary that is made of 10 µm-thick Lindemann glass (Hilgenberg GmbH) and filled with diamond microcrystals with nominal particle sizes of 3 µm (The Nilaco Corporation). The capillary is attached to the holder, which is mounted on two motor stages, with polyamide tape, such that the longitudinal direction of the capillary becomes horizontal. When using the spectrometer, the capillary moves to the X-ray beam path and the MPCCD detector measures one of the Bragg diffraction peaks in the vertical plane. The MPCCD detector is motorized to rotate around the capillary and covers the diffraction angle (2θ) ranging from 13.5 to 153.0°. The distance from the capillary to the MPCCD detector is 0.33 m. The data-acquisition rate of the MPCCD detector is set to the repetition rate of the XFEL pulse, and the detector is synchronized to each XFEL pulse.

From the differential form of Bragg’s law, the increment of the diffraction angle [δ(2θ)] is related to that of photon energy (δ*E*) by 

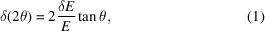

where *E* is the central photon energy of the XFEL pulse. With this equation, the single-shot spectrum can be evaluated from the peak shape of the powder diffraction profile. One can use the spectrometer simply by translating the capillary to the X-ray beam path and moving the detector to cover the expected diffraction angle. The alignment of the spectrometer does not need iterative procedures and is straightforward. Since the capillary absorbs the X-ray beam and distorts the wavefront of the transmitted beam, we employ this spectrometer solely for the machine tuning at present.

## 3. Energy resolution of the spectrometer

The energy resolution of the spectrometer mainly depends on two factors. One is the uncertainty of the diffraction angle 2Δθ, which is of the order of 100 µrad, due to the finite diameter of the capillary and the limited spatial resolution of the detector. This uncertainty limits the relative energy resolution (the energy resolution normalized by the central photon energy) of the spectrometer to be 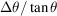 , which is of the order of 10^−4^. The other factor determining the energy resolution is the angular spread of the diffraction peak due to crystal strain and finite crystallite size. According to the literature by Williamson & Hall (1953 ▸), the angular spread of the diffraction peak may be expressed as 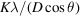 + 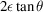 , with the Scherrer constant *K* ≃ 1, the X-ray wavelength λ, the crystallite size *D* and the crystal strain ε. The relative energy resolution of the spectrometer limited by the second factor can be expressed as 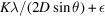 . A previous X-ray diffraction experiment of the adopted diamond crystals showed that the diffraction peaks are sharp even for high reflection indices (Nishibori *et al.*, 2007 ▸), indicating that the lattice constant of the crystals is highly uniform and ε is negligibly small. Since microcrystals enable suppressing the value of *K*λ/*D* to ∼10^−4^, the relative energy resolution limited by the second factor is also of the order of 10^−4^. The relative energy resolution of the spectrometer Δ*E*_spectrometer_/*E* may be expressed as 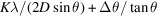 . Given that the typical energy spread of each XFEL pulse is a few tens of eV, the energy resolution of the spectrometer should be sufficient for evaluating fundamental spectral properties of XFEL pulses, such as central photon energy and photon-energy spread. Besides the above two factors, the energy resolution may be degraded when the number of crystals contributing to the X-ray diffraction is insufficient and the diffraction profile is distorted.

To experimentally evaluate the energy resolution of the spectrometer, we used XFEL pulses monochromated by the silicon(111) double-crystal monochromator in the optics hutch (Tono *et al.*, 2013 ▸) [the relative energy spread Δ*E*_mono_/*E* = 1.3 × 10^−4^ with full width at half-maximum (FWHM) photon-energy spread Δ*E*_mono_]. By measuring how much the photon-energy spread evaluated with the spectrometer deviated from the actual value, we estimated the energy resolution of the spectrometer. The single-shot spectra of the monochromated pulses were measured with the spectrometer and the average spectrum for 1000 successive pulses was calculated. From the FWHM photon-energy spread of the averaged spectrum Δ*E*_spectrum_, the energy resolution of the spectrometer was determined by 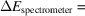 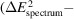 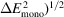 .

Fig. 1 ▸(*b*) shows the relative energy resolution of the spectrometer (Δ*E*_spectrometer_/*E*) as a function of the diffraction angle for typical photon energies of XFEL pulses at SACLA BL3 (6–12 keV). In the figure, each marker represents the energy resolution for a specific reflection index. Regardless of the photon energy, the energy resolution of the spectrometer improved with the diffraction angle. By selecting any one of the reflections with 2θ > 70°, one can realize the relative energy resolution much better than 10^−3^, which is sufficient to evaluate the photon-energy spread and the central photon energy of the XFEL pulses. The resolution of the spectrometer was consistent with the estimated value discussed above. In fact, the relative energy resolution of the spectrometer can be well reproduced by 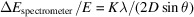 + 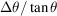 with *K*/*D* = 3.5 × 10^−4^ Å^−1^ and Δθ = 150 µrad [solid curves in Fig. 1 ▸(*b*)], indicating that the degradation of the resolution due to the limited number of crystals was not significant.

## 4. Examples of single-shot spectra and application of the spectrometer to machine tuning

**Figure 2:**
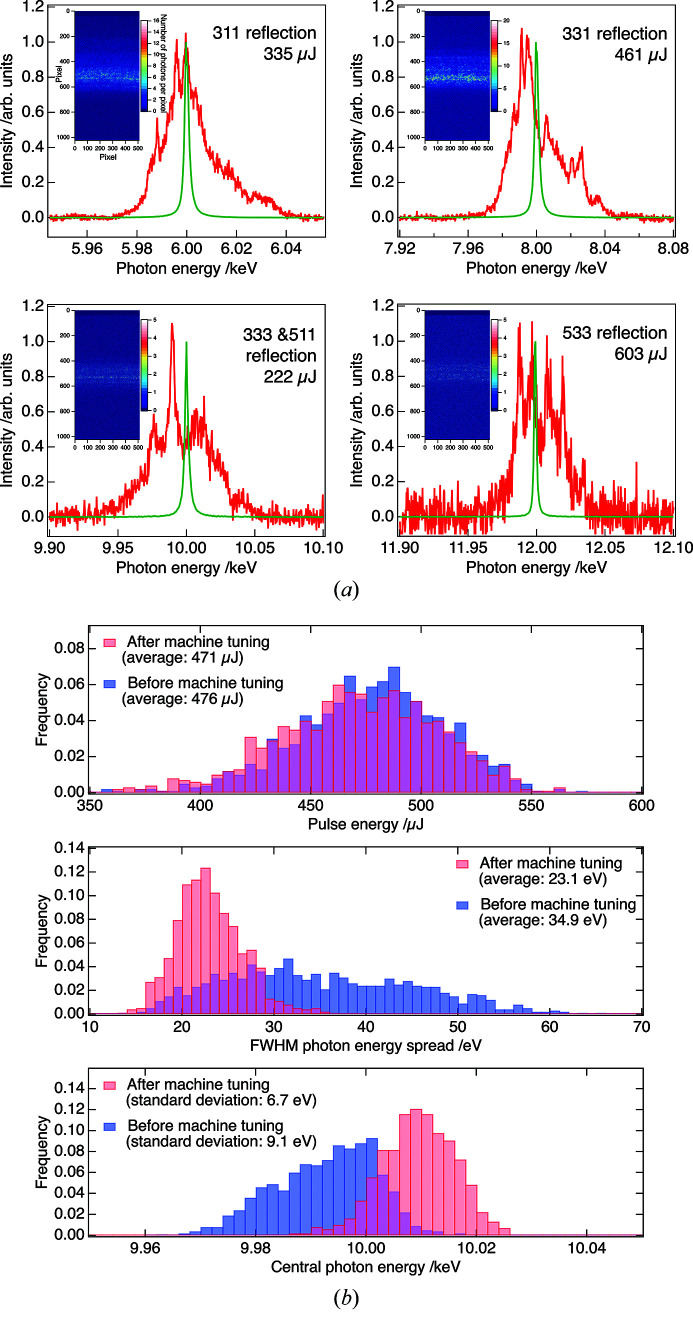
(*a*) Single-shot spectra of XFEL pulses measured with the spectrometer (red curves). Corresponding reflection index, pulse energy and diffraction image measured with the MPCCD detector are shown in each plot. Green curves represent average spectra of monochromated XFEL pulses (Δ*E*_mono_/*E* = 1.3 × 10^−4^) measured with the spectrometer. (*b*) Histograms of photon parameters (pulse energy at the optics hutch, photon-energy spread and central photon energy) of 10 keV XFEL pulses before and after machine tuning.

The developed spectrometer enables shot-by-shot evaluation of the spectral properties. Fig. 2 ▸(*a*) shows examples of measured single-shot spectra and corresponding diffraction images. The average spectra of the monochromated XFEL pulses measured with the spectrometer (see Section 3) are also shown for reference. The pulse energy shown in Fig. 2 ▸(*a*) represents the one measured with a calibrated intensity monitor at the optics hutch (Tono *et al.*, 2013 ▸), which is positioned upstream of the monochromator and the spectrometer.

This spectrometer plays an essential role in the machine tuning of SACLA. Recently, the SACLA facility has developed a machine-learning algorithm for daily machine tuning to maximize spectral brightness of the XFEL pulses while suppressing the shot-by-shot fluctuation of the central photon energy (Iwai *et al.*, in preparation). The spectral information about XFEL pulses obtained with the spectrometer is used as input parameters for the algorithm. Typical tuning time, including the setup of the spectrometer, is 30 min. Fig. 2 ▸(*b*) shows histograms of photon parameters (pulse energy at the optics hutch, photon-energy spread and central photon energy) for successive 1000 10 keV XFEL pulses (repetition rate of 30 Hz) before and after the machine tuning using the machine-learning algorithm. Here, the central photon energy and photon-energy spread were evaluated by fitting each single-shot spectrum measured with the spectrometer by a Gaussian function. Although the average pulse energy was almost the same before and after the machine tuning [476 µJ (before) and 471 µJ (after)], the photon-energy spread was considerably narrowed [average values of FWHM photon-energy spread were 34.9 eV (before) and 23.1 eV (after)]. Furthermore, the fluctuation of the central photon energy was suppressed by the machine tuning [standard deviations of the central photon energy were 9.1 eV (before) and 6.7 eV (after)]. The machine tuning using the spectrometer contributed to increasing the spectral brightness of the XFEL pulses.

## 5. Summary

In summary, we have developed a simple single-shot spectrometer using diamond microcrystals to diagnose the spectral properties of XFEL pulses. It was confirmed that the resolution of the spectrometer is sufficient for evaluating the central photon energy and the photon-energy spread of each XFEL pulse. This single-shot spectrometer has been installed at BL3 of SACLA and used for daily machine tuning.
